# Reconstruction of the Scrotum With Bilateral Gracilis Muscle Rotational Flaps in a Patient With Acute Myeloid Leukemia Complicated by Fournier’s Gangrene

**DOI:** 10.7759/cureus.25988

**Published:** 2022-06-16

**Authors:** Emily R Finkelstein, Federico Perez-Quirante, Zubin Panthaki

**Affiliations:** 1 Department of Surgery, Division of Plastic and Reconstructive Surgery, University of Miami, Miami, USA

**Keywords:** pelvic reconstruction, gracilis muscle flap, acute myeloid leukemia (aml), fournier, fournier gangrene

## Abstract

We describe the case of a 43-year-old male diagnosed with acute myeloid leukemia complicated by Fournier’s gangrene. Multiple debridements led to the complete effacement of the scrotum, with 360 degrees of exposed testes and a narrow base of suspension. It was decided to reconstruct the scrotum using bilateral gracilis muscle rotational flaps, followed by split-thickness skin grafting from the thigh. The gracilis muscle as a donor flap allowed for the protection and support of the testes and suspensory tissue while achieving an aesthetically pleasing result that resembled the normal scrotum. We hope providers will consider this reconstructive method in future patients who present with similar extensive effacement of the scrotal tissue.

## Introduction

Fournier’s gangrene is a rare type of necrotizing fasciitis of the perineum and genitalia. Infection is most often polymicrobial, arising from the colorectal or urogenital tracts [1,2]. The single greatest predisposing factor for disease development is diabetes mellitus, found in up to 66% of affected patients [2-4]. Other common comorbidities include alcoholism and obesity [2-4]. Despite recent advances in medical management, Fournier’s gangrene continues to carry a substantial risk of mortality, as high as 90% with any delay in care [1-6]. Therefore, early recognition and treatment with broad-spectrum antibiotics and surgical debridement are critical [1,3,6].

Patients that undergo multiple debridements can acquire sizable soft tissue defects including effacement of the scrotum, necessitating elective reconstruction [1,3,4]. Although there are many approaches to scrotal reconstruction described in the literature, they all share a primary goal of providing coverage to the exposed testes [7-9]. More recently, there has been increased emphasis on other goals of care such as preserving testicular function and achieving aesthetically pleasing results [4,7,8]. For those studies that describe some of these methods of reconstruction, few have documented cases that provide sufficient protection and coverage for 360-degree defects that include the exposure of the spermatic cord. This number is even less when considering a patient with hematological malignancy as the cause of their Fournier’s gangrene.

We present one of the few documented cases of Fournier’s gangrene attributed to acute myeloid leukemia (AML) in a 43-year-old male. Multiple debridements caused complete effacement of the scrotal tissue and exposed the spermatic cord. We reconstructed the scrotum in a single procedure using bilateral gracilis muscle rotational flaps, followed by split-thickness skin grafting over the testes. Achieving aesthetically pleasing and functional results, we hope that this case description provides new evidence behind using this type of muscle flap to recreate the scrotal sac and will guide physicians through this method of reconstruction. In addition, we anticipate this information will contribute to the limited existing literature on patients with Fournier’s gangrene initiated by AML [5,10].

## Case presentation

Consent was obtained from the patient prior to gathering information for this case report. The patient is a 43-year-old Honduran male diagnosed with AML in September of 2021 after going to the emergency department for symptoms related to COVID-19 infection. Laboratory evaluation while in the emergency department revealed a hemoglobin (Hb) of 4.1 g/dL, platelet count of 14,000, white blood cell count of 34,000, along with a positive COVID-19 antigen test. The patient was admitted to the hospital, and peripheral blood flow cytometry soon thereafter was indicative of AML. He was later started on induction chemotherapy with 7+3 D1 (cytarabine and an anthracycline antibiotic). Thirteen days following induction chemotherapy, his condition was complicated by neutropenic sepsis with *Steptococcous hemolyticus*, necessitating antibiotic treatment with cefepime, vancomycin, and metronidazole. The patient recovered and was discharged about two weeks later with a bone marrow biopsy showing morphologic remission.

About six weeks after his initial diagnosis, the patient was started on consolidation chemotherapy with HiDAC (high-dose cytarabine) C1D1 (day 1 of chemotherapy treatment cycle 1). He was managed as an outpatient before presenting back to the emergency department at the beginning of November complaining of severe fatigue, weakness, and diaphoresis for 24 hours duration. He also reported a tender mass in the left scrotum that had been progressively enlarging over four days. On arrival, the patient was hypotensive with tachycardia and tachypnea. Physical exam of the peritoneum revealed a 4x2 cm tender mass on the left inferior scrotum with minimal surrounding erythema. Labs were significant for neutropenia with a white blood cell count of 2.2 K/uL and lactic acid of 13.1 mmol/L. Ultrasound performed in the emergency department was consistent with bilateral orchitis with scrotal wall thickening and edema. Patient was then admitted to the medical ICU for treatment of neutropenic sepsis and started on a combination of vancomycin and cefepime. 

The next morning, the patient was found to have a temperature of 39 degrees Celsius, blood pressure of 100/62 mmHg, heart rate of 135 beats per minute (bpm), and respiratory rate of 31 breaths per minute. Physical exam of the perineum revealed severe induration and edema of the scrotum with necrotic skin overlying the left inferior scrotal tissue, findings consistent with Fournier’s gangrene. Patient was subsequently taken into emergent surgery for extensive debridement and wash-out of the wound. Cultures taken from the tissue of the perineum were positive for a heavy growth of *Escherichia coli *(*E. coli*). 

On postoperative day (POD) 13 from the initial debridement, the patient underwent a second surgery for further debridement of the perineum and subsequent reconstruction. Following this debridement, the scrotal tissue was completely effaced, leaving 360 degrees of exposed testes and a distal spermatic cord on a thin suspensory base. This posed a complicated case for reconstruction due to the complete loss of the scrotum, including the posterior, along with the relatively weak connection holding the testes in position. To avoid having to skin graft 360 degrees around the scrotum while accounting for the portion of the exposed spermatic cord, we decided to use bilateral gracilis muscle rotational flaps to overlay the testes and recreate the scrotum.

To begin the procedure, the patient was induced by anesthesia and placed in a lithotomy position and the surgical area was prepped and draped in the usual fashion. Fifteen-centimeter (cm) longitudinal incisions were created along the inner aspect of the thigh along the belly of the gracilis muscle. Once the muscle was properly identified, it was sharply dissected and separated from the surrounding muscles. The vascular pedicle was identified and preserved proximally, while secondary vascular pedicles were located distally and were divided using surgical clips. A second 5-cm incision was made distally, directly over the tendinous insertion of the muscle, followed by identification and division of the underlying tendon. At this point, hemostasis was evaluated and addressed by electrocautery. After achieving hemostasis, each gracillis muscle was tunneled under the groin and draped over each testicle. The flap dissection planes were closed using 2-0 Vicryl sutures that were placed over large Jackson Pratt (JP) round drains. 3-0 Vicryl was used to close the skin and reapproximate the underlying Scarpa’s fascia and deep dermis. Staples were used to superficially close each of the four incision sites. 

Transitioning over to the draped flaps covering the testes, the muscular belly was secured to each teste posteriorly using 3-0 Vicryl sutures. Superiorly, the pubic area was undermined, advanced, and sutured to the base of the penis, primarily closing the superior defect. Subsequently, a 15 by 10 cm split-thickness skin graft was harvested from the left anterior thigh after injection of 100 ccs of saline mixed with 30 ccs of 1% lidocaine with epinephrine. The harvested graft was meshed using a Zimmer Mesher in a 1 to 1.5 ratio. The skin graft was then draped and secured to the edges of the wound using 3-0 Chromic sutures. Several quilted sutures were placed to ensure the graft was secured to the surgical bed. Finally, the skin donor site was dressed using duoderm, and a wound vac with an underlying acticoat was placed over the flap site. 

**Figure 1:**
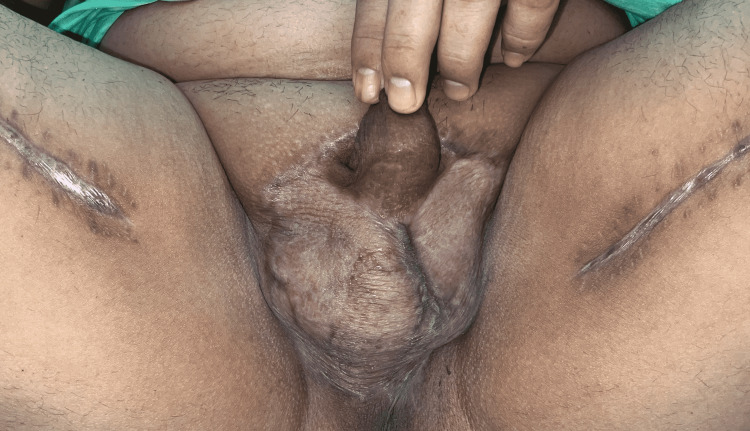
Patient photo taken during follow-up visit approximately three months after scrotum reconstruction

The patient’s postoperative course was insignificant. The site of the flaps healed quickly, allowing for the discontinuation of the wound vacuum on POD 7. Upon discharge on POD 13, the scrotum flap appeared minimally swollen, but overall was not bulky. The patient was pleased with the overall aesthetic results and did not suffer from contracture following recovery (Figure 1).

## Discussion

We present the clinical course of an immunocompromised 43-year-old male with AML complicated by Fournier’s gangrene that required multiple surgical debridements, resulting in extensive soft tissue loss. Subsequent reconstruction of the scrotal sac was performed utilizing bilateral gracilis muscle rotational flaps with split-thickness skin grafts from the anterior thigh. The patient made a full recovery with excellent functional and cosmetic results. 

A systemic review published in 2021 found only 44 documented cases of Fournier’s gangrene as a complication of hematogenous malignancy or its treatment, with only a fraction of them being attributed to AML specifically [5]. Two of the 44 identified cases underwent reconstruction using musculocutaneous flaps, with one being a pediatric patient, and both of whom died shortly after the operation [5,11,12]. Neither received bilateral gracilis muscle flaps as their method of reconstruction [5,11,12]. Although based on a finite number of documented cases, current evidence suggests that patients with hematological malignancies pose additional hardships in the management of Fournier’s gangrene [5-7,10]. Albasanz et al described increased rates of mortality in the population of patients with hematological malignancy [10]. Additionally, these patients may possess a higher risk of harboring nosocomial, multi-drug resistant organisms due to severe granulocytopenia [6,10]. Creta et al. found that more serious infection with monomicrobial *Pseudomonas aeruginosa *was more common in those with an underlying hematological disease when compared to the general population of Fournier’s gangrene patients [5]. Though our patient had positive cultures for *E. Coli*.

While there is no consensus regarding the best method for reconstruction of the scrotum following debridement, the approach can be partially determined by the extent of the tissue defect [4,8,13]. In defects affecting less than 50% of the scrotal tissue, possibilities for reconstruction include healing by secondary intention, subcutaneous thigh pockets, local scrotal advancement flaps, and split-thickness skin grafting [1,7-9]. When the defect spans more than 50% of the scrotum, options for reconstruction are limited to split-thickness skin grafting, fasciocutaneous flaps, or musculocutaneous flaps with or without the use of concurrent split-thickness skin grafting [1,7-9]. The use of a split-thickness skin grafting alone to cover perineal tissue defects is a well-described method in the literature, likely due to the relative simplicity of the procedure and a short recovery course [1,7,8,13-15]. Nonetheless, graft contracture is a feared complication of skin grafting to the testes, with the potential to cause severely restricted movement and substantial pain [4,7,8,13,15-17]. Graft contracture was an important concern for our patient, who would require a 360-degree spherical graft, paving the way for significant pain if contracture were to occur. The aesthetic results of skin grafting are mixed in the literature, with select studies reporting suboptimal outcomes [9,13]. It was decided that our patient was not an ideal candidate for this approach due to the thin, narrow base of suspensory tissue and exposed spermatic cord left anchoring the testes, which would not be well supported by a simple skin graft. 

Local or regional flaps have been a safe and effective method of coverage for severe tissue defects of the scrotum, without posing the same risk of tissue retraction as skin grafting [7,8,13,17]. When selecting a donor flap site, the functional, aesthetic, and psychological implications should be considered [4]. Suboptimal cosmetic results have been reported for flaps that are bulkier, such as the anterolateral thigh (ALT) flap [8,16]. Alternatively, cases describing the use of vertical rectus abdominis myocutaneous flaps (VRAM) and medial thigh flaps have resulted in outcomes that do not mimic the normal scrotal appearance, in part due to bulkiness [7,8,16,17]. In addition to poor aesthetics, flap bulkiness may also contribute to spermatogenesis arrest [18]. Wang et al described cases of thin trimming of scrotal may reduce their bulkiness while reversing the spermatogenesis arrest, allowing preserved sexual function [18].

There are many benefits to applying bilateral gracilis muscle flaps for the reconstruction of the scrotum [4,7,13,14,17,19]. In addition to providing sufficient coverage of larger and deeper defects, they offer numerous functional and aesthetic benefits [7,8,13,14,17,19]. The gracilius muscle is a well-vascularized donor tissue that may lead to better adaption to recipient surfaces and, ultimately, decreased rates of seromas and hematomas [8,14,17]. This vascularization may also provide increased resistance to infection, along with the ability to perform early reconstruction, leading to shortened hospital stays and improved quality of life [4,13,14,17,19]. Since the bulkiness of the donor muscle is often of aesthetic concern, it has been documented that muscle flaps in general tend to experience a great deal of atrophy following denervation compared to fasciocutaneous flaps [20]. With the expectation of significant atrophy from the gracilis throughout the recovery period, this donor muscle does not require debulking during the initial procedure, and more commonly results in thickness that resembles the natural contour of the scrotum [7,8,19].

Our patient recovered quickly and did not develop any complications following reconstruction. Upon follow-up, the testes were freely mobile in the reconstructed scrotal sac, which had atrophied to resemble a natural tissue of the scrotum. He was overall satisfied with the aesthetic appearance, with even further improvement expected in the months to come. Therefore, we implore surgeons to consider this reconstructive method in future patients presenting with 360-degree defects or narrow bases of suspensory support for the testes. 

## Conclusions

Debridement in the case of severe Fournier’s gangrene can lead to 360-degree effacement of the scrotum and loss of the tissue surrounding the spermatic cord, creating a narrow base of suspension. In our patient, reconstruction with bilateral gracilis muscle rotational flaps followed by split-thickness skin grafting yielded an excellent functional and aesthetic result. The gracilis muscles atrophied over a period of weeks, creating a scrotum that resembled the native tissue. Therefore, we encourage surgeons to choose this method of reconstruction for future cases presenting with 360 degrees of exposed testes with or without narrow bases of suspensory support.
